# Participatory mapping to address neighborhood level data deficiencies for food security assessment in Southeastern Virginia, USA

**DOI:** 10.1186/s12942-022-00314-3

**Published:** 2022-11-07

**Authors:** Nicole S. Hutton, George McLeod, Thomas R. Allen, Christopher Davis, Alexandra Garnand, Heather Richter, Prachi P. Chavan, Leslie Hoglund, Jill Comess, Matthew Herman, Brian Martin, Cynthia Romero

**Affiliations:** 1 Department of Political Science and Geography, Old Dominion University, 7012 Batten Arts and Letters, Norfolk, VA 23529 USA; 2 Center for Geospatial Science, Education & Analytics, Old Dominion University, Innovation Research Park Building #1, Suite 402, 4111 Monarch Way, Norfolk, VA 23508 USA; 3 Department of Political Science and Geography, Old Dominion University, 7020 Batten Arts and Letters, Norfolk, VA 23529 USA; 4 Virginia Modeling, Analysis, and Simulation Center, Old Dominion University, 1030 University Blvd, Suffolk, VA 23435 USA; 5 Master of Public Health Program, Eastern Virginia Medical School, P.O. Box 1980, Norfolk, VA 23501 USA; 6 School of Community and Environmental Health, Old Dominion University, 3112 Health Sciences Building, Norfolk, VA 23529 USA; 7 Food Science & Nutrition Program, Norfolk State University, 700 Park Avenue, Norfolk, VA 23504 USA; 8 Master of Public Health Program, Eastern Virginia Medical School, Brock Institute for Community and Global Health, P.O. Box 1980, Norfolk, VA 23501 USA

**Keywords:** Food security, Participatory mapping, Agency, Capacity building, Nutrition

## Abstract

**Background:**

Food is not equitably available. Deficiencies and generalizations limit national datasets, food security assessments, and interventions. Additional neighborhood level studies are needed to develop a scalable and transferable process to complement national and internationally comparative data sets with timely, granular, nuanced data. Participatory geographic information systems (PGIS) offer a means to address these issues by digitizing local knowledge.

**Methods:**

The objectives of this study were two-fold: (i) identify granular locations missing from food source and risk datasets and (ii) examine the relation between the spatial, socio-economic, and agency contributors to food security. Twenty-nine subject matter experts from three cities in Southeastern Virginia with backgrounds in food distribution, nutrition management, human services, and associated research engaged in a participatory mapping process.

**Results:**

Results show that publicly available and other national datasets are not inclusive of non-traditional food sources or updated frequently enough to reflect changes associated with closures, expansion, or new programs. Almost 6 percent of food sources were missing from publicly available and national datasets. Food pantries, community gardens and fridges, farmers markets, child and adult care programs, and meals served in community centers and homeless shelters were not well represented. Over 24 km^2^ of participant identified need was outside United States Department of Agriculture low income, low access areas. Economic, physical, and social barriers to food security were interconnected with transportation limitations. Recommendations address an international call from development agencies, countries, and world regions for intervention methods that include systemic and generational issues with poverty, incorporate non-traditional spaces into food distribution systems, incentivize or regulate healthy food options in stores, improve educational opportunities, increase data sharing.

**Conclusions:**

Leveraging city and regional agency as appropriate to capitalize upon synergistic activities was seen as critical to achieve these goals, particularly for non-traditional partnership building. To address neighborhood scale food security needs in Southeastern Virginia, data collection and assessment should address both environment and utilization issues from consumer and producer perspectives including availability, proximity, accessibility, awareness, affordability, cooking capacity, and preference. The PGIS process utilized to facilitate information sharing about neighborhood level contributors to food insecurity and translate those contributors to intervention strategies through discussion with local subject matter experts and contextualization within larger scale food systems dynamics is transferable.

## Introduction

Food security is equitable access to food including safe access to nutritious food that is preferable and meets dietary needs. Food insecurity, wherein access to healthy and affordable food sources is not equitable, presents a vexing problem that is strongly correlated with both socioeconomic factors and long-term health outcomes [1, 2]. Inequitable access to healthy food is a structural problem that varies by and within households across geographies (i.e. census tracts or zip codes).

Food insecurity is associated with proximity, availability, and affordability of healthy food and classified as high, marginal, low, and very low by the United States Department of Agriculture (USDA). Low indicates capacity to maintain eating patterns but with reduced quality. Very low means reduced intake because of financial or other limitations. Areas identified as low and very low food security are a public health emergency. The USDA considers about 2.2 percent of United States’ households at risk of food insecurity because they live over 1.6 km from a grocery store and do not own a car, which limits the capacity to access healthy food [3].

Food security remains a significant component in the ongoing systemic problem of health inequities, where food insecurity can exacerbate other health, social, and economic problems—particularly in vulnerable communities [4–7]. The health impacts where food sources are non-existent, extremely limited, or costly lead to poor health outcomes. Research demonstrates a higher risk of heart attack and stroke, higher rates of diabetes, obesity, and other chronic diseases, and lower life expectancy [8]. Food shortages can trigger serious health and mental health consequences for young children and families [9, 10]. Whether shortages are chronic problems or stemming from circumstances such as hazards, food shortage is considered an emergency because it is associated with stress and adverse long-term physical and mental health outcomes [11]. These costs extend beyond individual outcomes within vulnerable populations—with far reaching public health, social, and economic societal impacts [12]. In this regard, food shortages can lead to trauma responses that are prevalently observed in many low-income and minority communities [11, 13, 14].

Interventions beyond immediate supply of food assistance—as food banks and charitable organizations can rapidly proliferate—are needed [15, 16]. Critical need extends beyond the charitable supply to address the underlying issues through political will by fully acknowledging the problem and taking effective steps to address the structural causes of health inequities, such as food insecurity, which remain in high-income countries with developed economies [12]. Policy and practical interventions that are both trauma-informed and stakeholder driven provide opportunities to tailor solutions to address root causes, align nutrition and access with culture and preferences, as well as, specific dietary needs, leverage the capacity of charitable organizations, and address supply and distribution problems [17].

Early market-based methods to spatially measure and address food security using Geographic Information Systems (GIS) do not produce wholistic assessments and interventions for complex, systemic issues related to food security. For example, the term food desert that became popular in the 1990’s for areas with low proximity to food options over-emphasizes the role of distance in behavior and decision-making [18]. Solutions for food deserts typically entail adding healthy food sources where they are scarce; however, these do not address temporal, economic, cultural, mobility, or other resource constraints that reduce access to healthy food even if it is in proximity. Research shows that opening a supermarket with fresh food does not directly translate to healthy diets or improved health outcomes without addressing structural disparities, such as housing, health, and employment [19–22]. Consequently, Widener [23] called for the term food desert to be retired in the late 2010’s to reduce the arbitrary nature of strictly spatial solutions.

Food desert research began to illuminate deficiencies of commercial business listings and other large-scale traditional datasets utilized in national food security assessments, such as those produced by the USDA and Center for Disease Control (CDC). Ground truthing also shows accuracy errors and incomplete listings particularly for sources smaller than supermarkets [24, 25]. Improvements in data availability and the development of interactive online platforms to conduct participatory GIS (PGIS) allow for dynamic modeling utilizing an increased set of variables across geographic scales and time in consultation with stakeholders to represent and contextualize food insecurity more accurately [26–28]. PGIS offers the opportunity to both improve spatial data used for food security assessment and engage stakeholders in a participatory process to collect qualitative data that informs wholistic, collaborative policy approaches.

Minimal data and documentation exist regarding the food access condition of socio-economically depressed neighborhoods in Southeastern Virginia. This pilot study of three cities in Southeastern Virginia (i.e. Norfolk, Portsmouth, and Chesapeake) goes beyond generalized geographic and socio-demographic indicators of food insecurity found in the existing literature to engage subject matter experts in food insecurity risk identification and area-specific intervention planning using PGIS. The objectives are two-fold (i) to improve upon foundational data that spatially quantifies food equity, security, and disparities using a participatory mapping process at the neighborhood scale and (ii) to examine the relation between the spatial, socio-economic, and agency contributors to food security using this granular spatial analytic. This technique provides insight into neighborhood-level food environments beyond traditional food sources (e.g., food banks and farmers markets) and establishes a transferable process grounded in food system dynamics to collect nuanced information for neighborhood scale interventions that improve health issues such as obesity, malnutrition, type II diabetes, and cardiovascular disease.

## GIS applications for food security assessment

GIS is the most utilized form of food security assessment. The evolution of GIS applications to food security has expanded assessment from spatial analysis of proximity to a participatory process triangulating spatial, socio-economic, and social networks. These changes reflect a more nuanced understanding of contributors to food insecurity and require more granular data collection techniques to further progress the field [29]. A review of these developments and opportunities follows.

In measuring proximity, GIS can use network topology to accurately estimate the time and distance for the surrounding population to reach a food source. It can also determine the population density reliant upon a particular food source [30]. Studies of food swamps add the type of food available, healthy or unhealthy, to proximity analysis but share similar issues as food desert research by only accounting for spatial factors [31, 32]. These market-based assessments may inform supply and demand and business siting but are largely divorced from resource and behavioral influences and best practices to improve health outcomes [19–22]. In particular, the right system of food sourcing and social interaction networks are needed for a new food source to meet community needs [3, 33].

Co-locating food sources not only with residences but also places of employment, healthcare, schools, and other businesses or services can be beneficial but is not usually the primary consideration in opening new locations [34, 35]. This disconnect makes individuals and households more reliant upon vehicles to access the full set of services desired or required for their health and well-being. Mobility, in terms of the mode of and capacity to travel expands upon proximity analysis through more realistically measuring time traveled. For example, public transit riders may have increased travel times and reduced hours in which to access transportation to healthy food sources [36].

Cumulative opportunity analysis of access to multiple healthy food options rather than just one has been shown to corelate with improved health outcomes [36]. Incomplete nutritional terrain assessment may compound health, accessibility, and affordability issues. For example, limited access to healthy food contributes to obesity and heart disease, which may cause reduced mobility and limit economic potential [30, 37]. Employment and household composition also modify the temporal, economic, and spatial components of food access. For example, employment may influence an individual to shop at a store closer to their place of work, with pricing appropriate for their income, or open before or after their shift [33, 38]. Similarly, household characteristics, such as having a child in school within a household may shift food source accessibility.

### GIS data inputs and analysis

Indices have been developed to spatially analyze multiple aspects of vulnerability to food insecurity, such as the Healthy Food Availability Index (HFAI) [39]. Various indices utilize different sets of factors depending on the intended output, which can be weighted to indicate the degree of influence for each factor in a given area. There are known issues with many national data sources input into indices. For example, commercial supermarket databases have known accuracy and completion errors, particularly exclusion of small community stores and lag in accounting for closures [24, 25]. Despite these known errors and limitations as a market-based tool relying on proximity, the USDA Food Access Research Atlas remains the most utilized index for food security assessment [18]. Vulnerability indices are limited both by the data inputs and non-transferable weighting systems that require community input to reflect local conditions. For example, findings in urban settings do not necessarily apply to rural settings. Bower et al., [1] study poverty and racial influences in the United States finding that both were independently indicative of food insecurity and low-income racial minorities experienced compounded effects in urban areas, but the same association is not present in rural areas.

Scale also limits the utilization of national level food and health data for local intervention because of the large areal units used to preserve the confidentiality of small population samples that compose Census tracts, block groups, or blocks. Jurisdictional misalignment between local government and Census groupings, such as cities or counties that include pieces of multiple tracts or block groups cause challenges in scaling data for intervention. Further, Modifiable Areal Unit Problem (MAUP) is inherent to coarse scale Census enumeration polygons versus the finer scale problem awareness community consultation produces [40, 41]. Although there are ways to down-scale national data, they require ground truthing. For example, micro-scaled data simulations in Detroit, Michigan show that local obesity rates overlapped more with USDA low-income tracts and CDC less healthy food tracts than USDA food desert tracts [42].

### Participatory GIS

PGIS increases granular data collection applications and means for community engagement, thereby decreasing known errors in coarse scale data and diversifying data contributors, especially through online web-based mapping [39]. De Master and Daniels [26] show that PGIS in conjunction with critical mapping techniques improve the understanding of food security by integrating community assets and contextualizing the role of proximity and transportation in food insecurity. For example, the Place-Based Food Insecurity and Vulnerability Index offers a means with which to unite multiple quantitative factors with qualitative understandings of local food systems using a PGIS to improve cultural responsivity and applicability for communities and households with the most at stake [39, 43].

Calls for resident driven processes to improve equity in food security priorities benefit from a variety of PGIS techniques [44]. Shannon [18] promotes the utilization of food diaries, qualitative interviews, Global Positioning Systems, and georeferenced photography to account for perceived access, quality, and transportation options to produce and unite behavioral, mobility, and other food security data. Douglas et al. [27] states that these PGIS methods are especially well suited to link social determinants of health with place-based policy intervention aligned with community connectivity including agency found in social and governance networks rather than geographic delineations. For example, food diaries and participant interviews in Cuba captured the role of household and community social networks in accessibility [45]. Indeed, social capital plays a critical role in health behavior that needs to be included for effective intervention [46]. For enduring structural change, the way that chronic diseases and food choices are addressed requires modification by cultivating a sustainable culture of health around food provisioning [18, 47]. Collective action and coordinated advocacy efforts reduce marginalization and restore a sense of place through leveraging resources and improving socio-economic conditions. Personal connections and partnerships both traditional and non-traditional facilitate asset redistribution and increased representation within communities [48, 49].

An organized framework to both describe and guide neighborhood level PGIS application is needed [28]. The International Fund for Agricultural Development developed best practices, but George and Timer [50] identify that they have not been adapted to individual country contexts through local pilot studies. This study frames results from its engaged research in the context of food security assessment to provide practical data expansion methods and inform interventions that can be used to make food distribution systems more tailored to vulnerable populations and make public health practitioners, government officials, and planners more aware of geographic, cultural, and socio-economic factors and networks contributing to food related health outcomes at the neighborhood level.

## Methods

The study uses a mixed methods approach to provide relational context to objective spatialized food access data [37, 45]. Similar to Kamel Boulos and Koh’s [51] *Smart City Lifestyle Sensing for Well-Targeted Public Health Intervention—Process Flow Diagram* this participatory mapping approach fosters information sharing between stakeholders to inform interventions that improve overall food security. Scientifically accepted indices and data analysis processes, such as the HFAI [39] and the *Living Future Baltimore City Food Environment Report* [44], were utilized to improve upon USDA aggregate data showing low income, low access [52] areas where fresh affordable food is limited and explore which contributors to food insecurity affect neighborhood level unmet need. Results were interpreted in the context of national and international food systems literature to determine applicability and transferability of the PGIS outputs and process.

### Study area

**Fig. 1:**
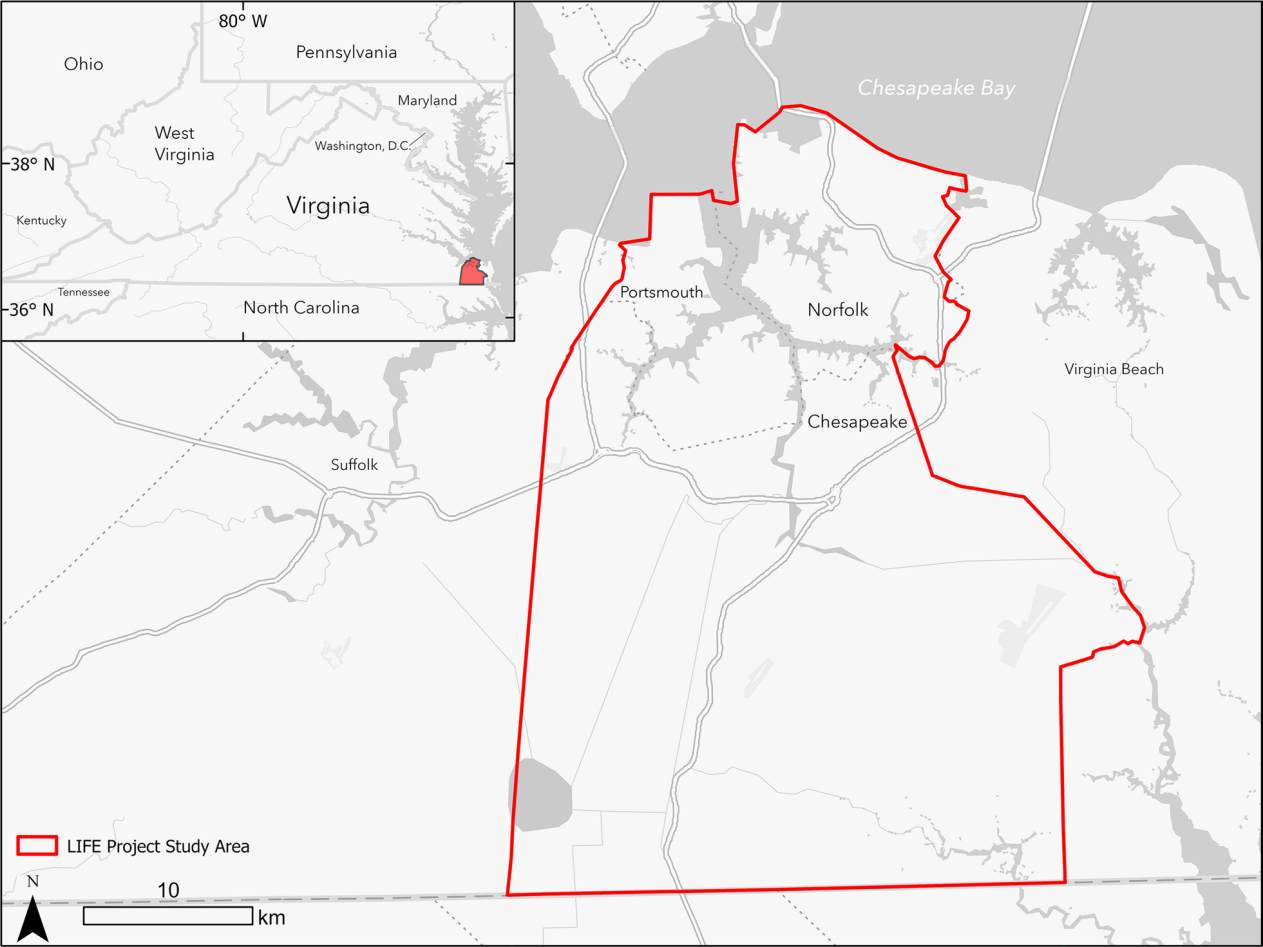
Study area: Southeast Virginia cities of Norfolk, Portsmouth, and Chesapeake (Generated by authors)

The cities of Norfolk, Chesapeake, and Portsmouth, Virginia (see Fig. 1) include a diversity of rural and urban neighborhoods that span a broad range of socioeconomic and demographic conditions. Southeastern Virginia features the leading port by tonnage on the United States east coast. In 2020, the three cities were home to more than 0.5 million people (i.e. 238005 Norfolk, 97915 Portsmouth, and 249422 Chesapeake) living in approximately 1278.9 km^2^ (i.e. 249.4 km^2^ Norfolk, 120.7 km^2^ Portsmouth, and 908.8 km^2^ Chesapeake) [53]. The three coastal cities range in elevation from 2 m in Chesapeake to 5 m in Norfolk and are both subsiding and encountering higher than average rates of sea level rise making them prone to flood hazards from tides, tropical storms, and nor-easters [54–56].

More than one in three households qualified as asset-limited, income constrained employed households (i.e. income above the Federal Poverty Level but below the basic cost-of-living threshold), indicating the presence of socially and economically vulnerable populations in the region [54]. In 2019 the Foodbank of Southeastern Virginia and the Eastern Shore [57] identified 45010 food insecure individuals in Norfolk, 17750 in Portsmouth, and 25870 in Chesapeake. Social isolation, limited childhood opportunities to learn about food security and finances, negative life events, and competing budgetary priorities are the root causes for food insecurity in this area. Socio-economic factors associated with seeking emergency food assistance from community-based organizations that supplement welfare programs included age, race, number of adults and children in the household, housing and employment status. Residents’ lifetime experience combined with fragmentation in the welfare system reduces capacity for poverty alleviation [57].

### Participant recruitment

In January 2022, researchers at Old Dominion University, Eastern Virginia Medical School, and Norfolk State University convened an online focus group event “Location Intelligence for Food Equity (LIFE): Identifying Access to Healthy Food Choices in Hampton Roads” using geospatial technology to address the limitations of existing geospatial food data in identifying availability and unmet need through a participatory research process. The focus groups utilized a targeted snowball sample of 51 professionals working with food insecure communities in Norfolk, Chesapeake, and Portsmouth, Virginia derived from researcher contacts. Invitations were sent via email with a request to forward to additional relevant contacts. Thirty-two participants registered through Eventbrite for the focus group. Of these registrants, 29 participated in the focus groups. Participants reported their professional expertise as follows: 8 food distribution, 10 nutrition management, 8 human services, 2 related research, and 1 not provided. Participation was voluntary. All registrants received focus group materials via email.

### Focus group agenda

The focus group was hosted on Zoom for 1.5 h with time allocated as follows: introduction, 10 min; break-out session for data collection, 75 min; and 5 min closing remarks. Data collection involved three parts of 25 min each (i) food sources, (ii) areas of unmet need, (iii) discussion. Three predetermined questions guided each part [58]. Food source questions included: *1. Where are additional food sources not already visible in the map? 2. Which of these include healthy food? Describe food options available. 3. Which do not include healthy food? Describe food options available.* Areas of unmet questions included: *1. Where are areas of unmet need for healthy food? (rank low–high) 2. Where are accessibility challenges that reduce access to healthy food sources? 3. Does the neighborhood food environment allow food access for health management, or do limited hours of operation, affordable food prices, healthy foods availability, public transportation options, safe walkways and sidewalks, proper lighting, security, *etc*. reduce access?* Discussion questions included: *1. What other resources do you find useful in determining food security? Please share data and links as possible in the chat or verbally with us. 2. How can additional resources be leveraged and delivered in a timely manner? 3. Are there any additional thoughts you would like to share or questions that should be asked?*

### LIFE webapp

**Fig. 2:**
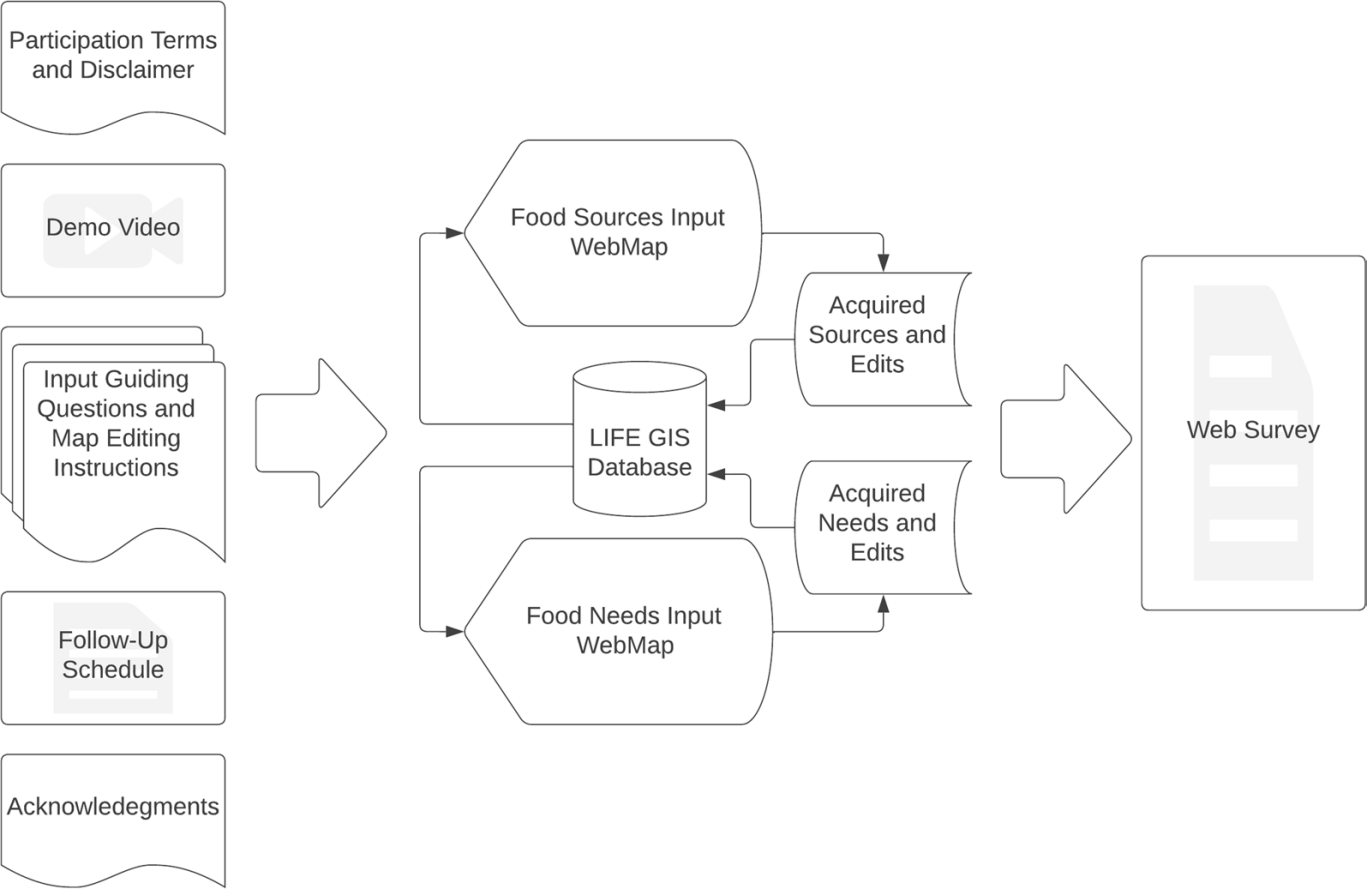
LIFE Storymap Diagram. Left: Tabs scrolled through by users in consecutive order from top to bottom. Center: Embedded LIFE Webapp input and editing procedure to establish LIFE GIS database Right: Embedded Qualtrics survey (Adapted by authors) [58]

The LIFE Webapp and associated storymap interface [58] was developed as a participatory mapping aid to the focus groups. The ArcGIS StoryMap through which participants and facilitators could input GIS and survey data included a disclaimer to ensure voluntary participation and identity protection, a training video, written instructions to guide users through the data collection process, a follow-up schedule to indicating when participants should expect to receive study outputs, and funder acknowledgements. Figure 2 shows a diagram of data collection process available through the storymap that produced the LIFE GIS database.

The application used Esri’s Web AppBuilder application programming interface as its platform. Data layers showed population density, transit options, and food sources at the regional, and local scales through the Layer List menu. Table 1 lists the map layers, key attributes, and data sources. Layers shown to the focus group participants were pulled in through the Esri ArcGIS Online interface via web feature services from authoritative data sources such as the 2020 US Census, the Foodbank of Southeastern Virginia and the Eastern Shore, the Social Vulnerability Index, and Esri’s ArcGIS Business Analyst, which uses North American Industry Classification System codes to identify businesses through their partnership with SafeGraph, a data company that provides demographic, advertising, real estate, and other data. Following the methodology of Misiaszek et al. [44] neighborhood food sources, such as small stores, public, virtual, farmers markets, nutrition assistance programs, and urban agriculture, were displayed in addition to traditional large grocery stores.

**Table 1:** Webapp Map Layer Characteristics and Sources (Adapted by authors) [58]

Layer name	Characteristics	Source
Norfolk, Portsmouth, and Chesapeake K-12 Public Schools	Point locations for K-12 public schools	Hampton Roads Geospatial Exchange Online (HRGEO): K-12 Schools [59]
Grocery and Other Market Locations	Point locations for grocery store and market locations	ArcGIS Business Analyst [60]—Business and Facilities Search for Food Service Locations and Grocery and Other Locations by city using SafeGraph
Fast Food Locations	Point locations for limited-service restaurants	ArcGIS Business Analyst [60]—Business and Facilities Search for Food Service Locations and Grocery and Other Locations by city using SafeGraph
Convenience and Corner Store Locations	Point locations for convince and corner store locations	ArcGIS Business Analyst [60]—Business and Facilities Search for Food Service Locations and Grocery and Other Locations by city using SafeGraph
Foodbank and Food Pantry Locations	Point locations of addresses—Food Pantry Directory	Foodbank of Southeastern Virginia and the Eastern Shore [61]
Missing Food Locations	Point locations input based on LIFE Workshop	Participant input [58] and Healthy Chesapeake [62]
Areas of Need	Polygon layer for input based on LIFE Workshop	Participant Input [58]
Population	2020 U.S. Decennial Census—block level data	ArcGIS Online Living Atlas [63]
CDC Social Vulnerability Index	Social Vulnerability Index [64]—Socioeconomic Theme tract level data	CDC / Agency for Toxic Substances and Disease Registry
Hampton Roads Transit Routes and Bus Stops	Public transit network	HRGEO: Hampton Roads Public Transit Routes [65], HRGEO: Hampton Roads Public Transit Stops [66]
Low Income and Low Access Census Tracts at 1 and 16 km distance from a Grocery Location	Food Access Research Atlas [67]	USDA

The USDA Food Access Research Atlas [67] layers for low-income, low-access were consulted for reference information as a proxy for proximity, accessibility, and affordability. The USDA layers polygons were shown to participants to indicate expected food insecurity based on income and distance to a recognized large food source, such as a supermarket or grocery store included in the Store Tracking and Redemption System or TDLinx. Of note, these sources are known to provide a proxy rather than a full set of stores with healthy food [52]. Further, data input and analysis are not real-time, potentially featuring a year or more year lag for American Community Survey Data or other updates in which socio-economic and store locations could change. For example, the Food Access Resource Atlas used from federal to local government for planning and public health initiatives can be up to 5 years old [68].

Two additional layers were derived from focus group input to compose the LIFE GIS Database including current store locations and socio-economic conditions indicating food insecurity at the neighborhood level. Participants identified sources by address in one layer within the categories of grocery and other markets, fast food, convenience and corner stores, foodbank or pantry, K-12 schools, and other and areas of need no smaller than a city block in another layer by type of accessibility issue including limited affordability and availability of healthy foods, public transportation, safe walkways, or other. Facilitators guided participants to utilize HFAI indicators to self-determine healthy and unhealthy designations based on their experiences in a given store [39]. A description could be added as an attribute to all feature inputs, as well as a ranking (low, medium, high) for the level of concern in an area of unmet need. Facilitators had the option to show or hide each layer throughout the focus group to assist participants in visualizing and assessing source inclusion and need. Finally, a discussion followed the identification of non-traditional food sources and areas of unmet need to foster community-centered interventions as suggested by the food topography approach to be needed for nuanced improvement planning [44].

Participants were randomly assigned to breakout groups of about equal size. Participants were asked to contribute geospatial feedback by instructing the breakout room facilitator from the research team to edit by creating new features (i.e. place points and polygons) on the maps to designate missing locations and areas of need. Focus group facilitators were provided basic tool functionality such as being able to zoom in and out on the map, search for a particular location, access the legend that shows data symbology, and the ability to turn on and off the various data layers using the layer list provided. The following instructions were provided for editing the Webapp:

To add a point to the map, click the Edit button on the left tab, then click Add feature. Place the point on the map at the location you think an additional food source should exist. Once you have placed a point on the map, you will be prompted to fill out some information about it. Under the NAME section, give your point a name (i.e. what you think should be there). Next under the FoodType section, select from the drop-down menu what type of food location your point is, and then finally under the Notes section list any additional information you would like us to have about your point. When you are done adding information, click Add to finish.

Use the same method as described above to add a polygon area to the map. You will need to draw all four sides of your area. Double click to close your area. Once you've drawn your polygon on the map, use the Rank section to assign the level of need to your area (low, medium, high). Then use the AccessibilityChallenges drop down menu to select the accessibility challenge that reduces access to healthy food sources for your area. Finally, use the Description field to add any additional information, for instance if you think there are multiple accessibility challenges [58].

Quality control was conducted to remove duplicative or inaccurate inputs (i.e. features and attributes) from the digital inputs to the webapps after the focus group. The following instructions were provided for deleting duplicative features: *To delete a point you have added to the map, click on the Edit tab, click Edit feature, select the point you want to delete, and then click the delete button at the bottom* [58].

### Data analysis

Focus group mapping and discussions were recorded and analyzed using a mixed methods approach. Layer attribute tables were utilized to produce descriptive statistics. Discussion transcripts were manually coded by two research team members based on themes that emerged from the responses to identify relationships between them. Descriptive quotes were identified and extracted to further explore participants’ concerns. Triangulation was conducted across qualitative, geospatial data sets.

## Results

Findings connect data disparities with a community perspective of contributing factors and actionable recommendations. A nuanced spatial and socio-economic understanding of neighborhood level food security is presented in light of existing and needed connective agency.

### Additional food resources

**Fig. 3:**
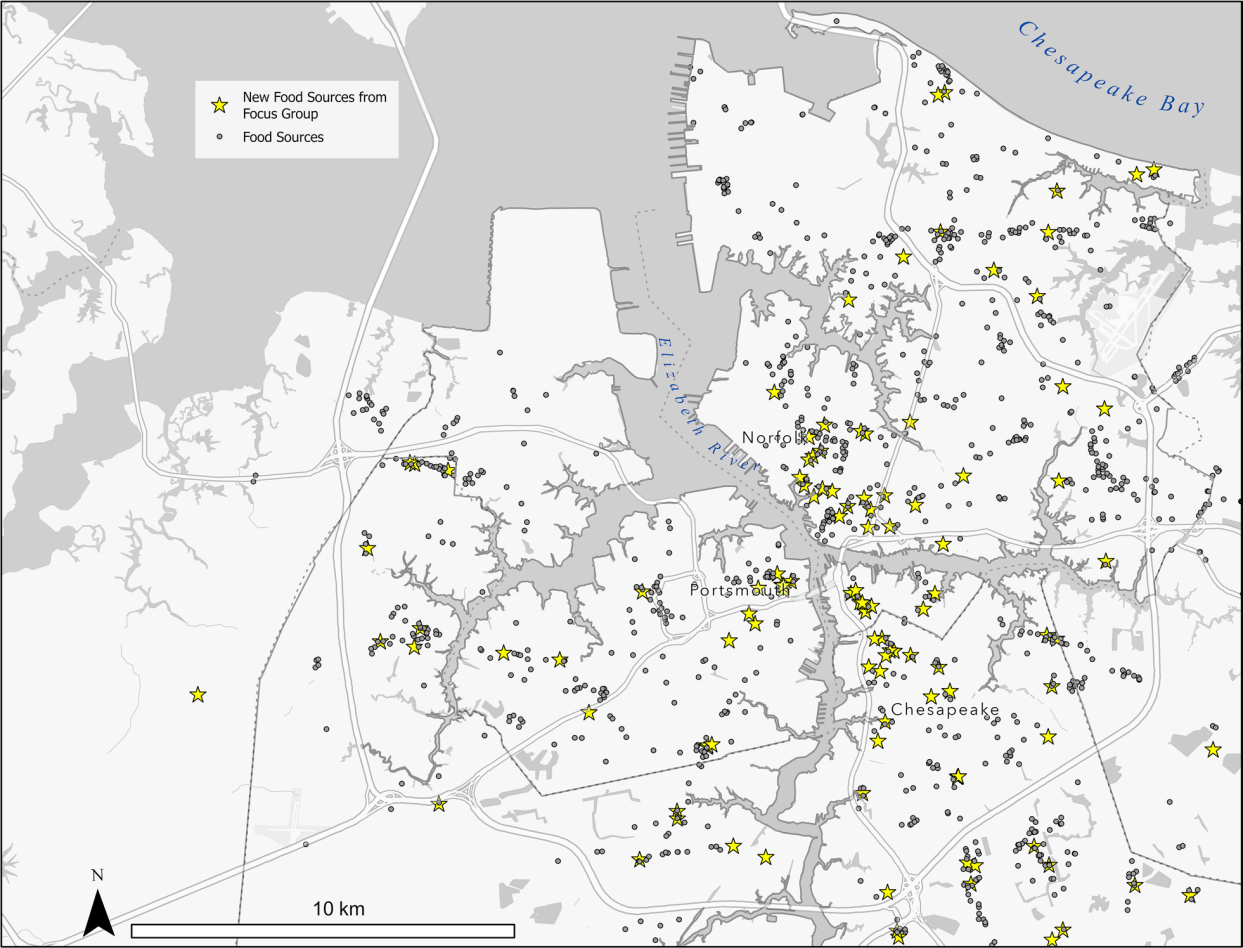
Food sources webmap. *Location attributes including name and food type are accessible in the LIFE storymap interface *[58] (derived from ArcGIS Business Analyst [60], Food Pantry Directory [61], and participant inputs) [58]

Food source data presented during the workshop did not include all local sources. Some sources were missed during initial data collection due to the data not existing on a public layer or outdated sources not providing correct current information. Figure 3 shows added sources. Almost 6 percent of total food sources and over 15 percent of healthy food sources were missing from the original dataset. Table 2 shows the participants identified missing source counts. An additional 72 points, 68 healthy and 4 unhealthy, were added to the 1145 from pre-existing sources, 383 healthy and 866 unhealthy sources. Additional food sources included community gardens, seasonal or pop-up markets, mobile and programmatic services, food pantries not connected directly with the Foodbank, CDC projects, education locations other than K-12 public schools, and community centers, nonprofits, non-traditional locations, and faith-based organizations that provide meals.

**Table 2:** Food source counts (Adapted by authors) [58]

	Unhealthy	Healthy	Total
Participant identified	68	4	72
Original dataset	383	866	1145

Non-traditional locations contributed to the discrete data collected. Prisons, the Chesapeake Health Department, schools, and other non-traditional locations operated community gardens. In some instances, ownership of the garden was transferred to the community once established, as was the case with those established by Bon Secours Medical Center. Community fridges (i.e. refrigerators) offered free groceries outside restaurants, such as the one outside Mea Culpa restaurant. A farmer was also reported to deliver food to Norfolk from a farm in the nearby city of Suffolk. Mobile programs, such as Youth Earn & Learn, provided food in varied locations or drop-off options. CDC programs, such as VA Investment Fund Grants, also brought healthy food options to small community markets and allowed new locations to open, such as Turners’ and St. Pauls’ markets in Norfolk.

Meals were provided at locations not traditionally categorized as contributing to the food mosaic. Nonprofit organizations, including The Boys & Girls Clubs, the Salvation Army, and homeless shelters, as well as childcare and early education centers, and private schools that prioritize underprivileged groups, such as Park Place, were added because meals are served. Head Start, Childhood Adult Care Food Program (CACFP), and backpack programs that make food available through schools and childcare centers were also added.

Rapid turnover and integration of long-standing programs in Norfolk were explained during additional data collection. Participants shared local knowledge that the Ghent Grab & Go, though not typically categorized as having healthy food because it is a convenience store, will have vegan and international options after planned refurbishing in 2022. Also, during COVID-19 a hotel was converted into a homeless shelter that included meals. Further, access to food programs expands as students can start in or progress through emerging education opportunities. A participant explained, “health food education is needed in elementary school education […] not short-term, but long term and improve selections when healthy food is available.” For example, YELLOWHAB opened in 2021 for low-income students. The Hague School has also been expanding the grade levels offered from 2019 through 2023. Changes in leadership at the Ghent Montessori School may create opportunities for new educational and outreach approaches, as well.

Food resource lists and nutritional support programs available for all three cities were considered by participants to be more current. Public food resource lists with local information included Foodbankonline.org, ABBA List, 211.org, Unite Us, Resources757.org, and City Virginia Beach and Chesapeake Social Services. Nutritional and health support programs existed through Anthem health insurance and Sentara hospital grants, Bon Secours hospital after care, nonprofits connecting backyard gardens to food insecure individuals, and Food Farmacy which fills prescription diets in Chesapeake. Additional national data sets including those held by Feeding America, the Department of Housing and Urban Development and the Virginia Department of Health’s CACFP were suggested for further data collection. Hospitals and providers that maintain health management records were another potential source of decentralized data.

Participants called for one compiled, consistently updated, accessible dataset. It was noted that the hosting requirements may be extensive, but there are examples of ongoing efforts to address these concerns with changing and missing data [69].

### Unmet need

**Fig. 4:**
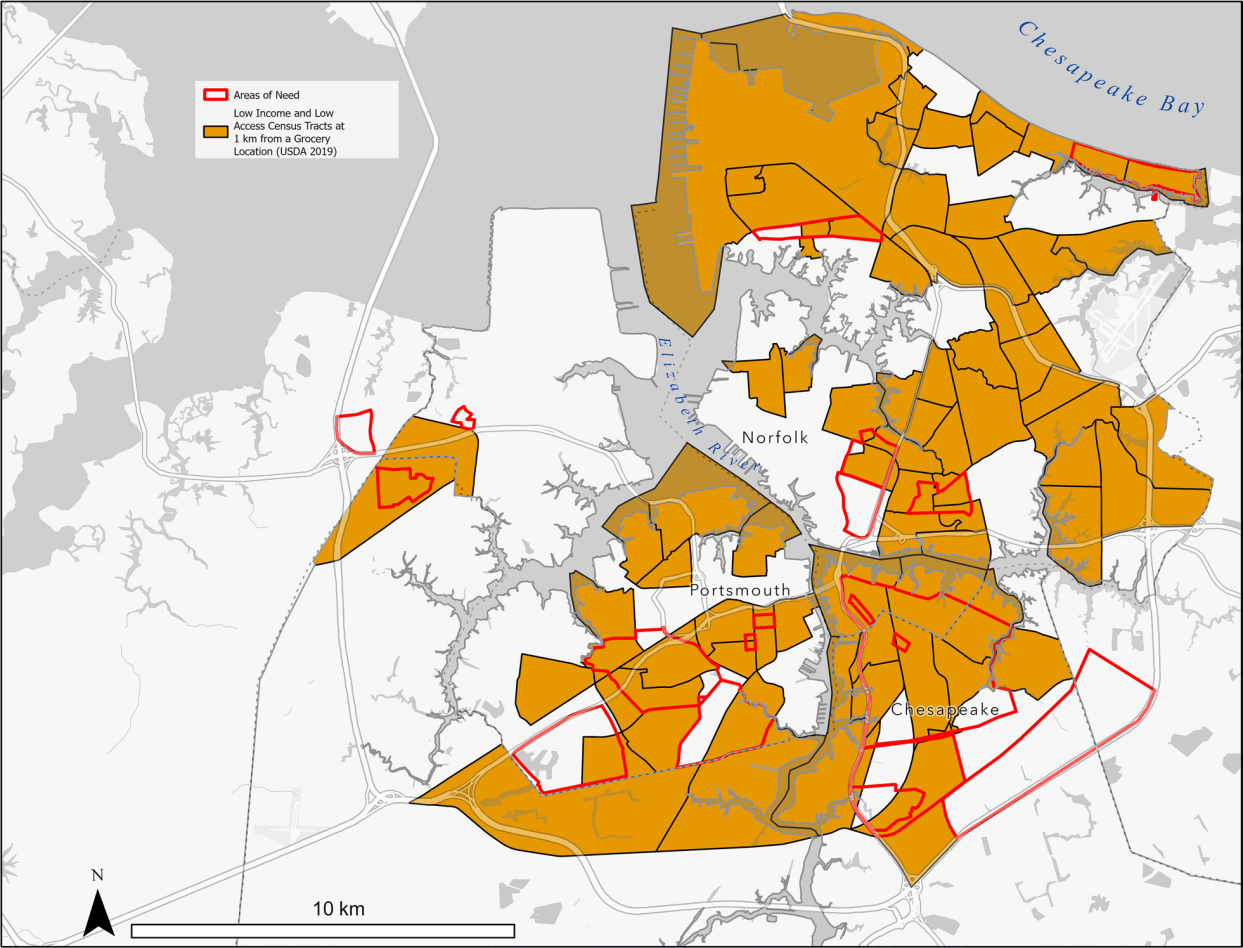
Food needs webmap *Polygon attributes including priority rank and accessibility challenge are accessible in the LIFE storymap interface *[58] (Adapted by authors from USDA Food Access Research Atlas [67] low-income/low-access Census tracts and participant inputs [58])

Figure 4 shows the areas with unmet food needs added into the dataset during the workshop. Participants identified 20 areas of need totaling 52.91 km^2^. Of that need 28.83 km^2^ overlapped with and 24.09 km^2^ was outside the USDA data. With a few exceptions, participant input on food needs largely agreed with the USDA Food Access Research Atlas’ [67] low-income, low-access tracts. Nonetheless, after data correction, there were new areas identified by participants that the USDA layer omitted or are a consequence of spatial aggregation [40, 41].

Table 3 shows accessibility challenges and priority rankings identified within areas of unmet need. Participants ranked 65 percent of the areas they identified with unmet need as high priority. Accessibility challenge associations indicated that contributors to unmet need were more complex than public transportation or safe walkways. Limited affordability and availability of healthy foods was the primary accessibility challenge attributed to 70 percent of the areas of unmet need.

**Table 3:** Areas of Unmet Need Rankings and Accessibility Challenges (Adapted by authors) [58]

	Limited affordability and availability of healthy foods	Public Transportation	Safe Walkways	Other
High	10	2	1	0
Medium	2	1	0	1
Low	2	0	0	0

Table 4 shows the healthy and unhealthy food source counts. Over 65 percent of food sources within areas of unmet need were unhealthy, which is 10 percent lower than the percentage of unhealthy food sources in the whole map and 11 percent lower than that in the USDA layers. While households may access healthy food sources outside identified areas of need, the comparatively reduced percentage of unhealthy food sources in areas of unmet need indicates that neighborhood level contributors are more complex than food source type distribution.

**Table 4:** Healthy and Unhealthy Food Source Count by Area Type (Adapted by authors) [58]

	Healthy food source	Unhealthy food source
USDA 0.5 Miles	174	349
Areas of Need	80	102
Whole Map	451	870

Participant’s qualitative inputs explored roles of connections between affordability, availability, transportation, walkability, and distribution. The availability of funds for food was linked to transportation limitations, the digital divide, and limited child-care options. Transportation may be needed to pick up SNAP and Special Supplemental Nutrition Program for Women, Infants, and Children benefits, which prevented some families from utilizing them even if they are eligible. Further, to be eligible for benefits, classes were required, which may also be difficult to attend with limited transportation. Similarly, involvement in Head Start programs at schools may be limited by transportation issues. Although food programs at schools were free to everyone in 2022, not everyone in need is taking advantage of them. Other economic concerns were associated with cost of healthy foods and utility of the federal assistance programs, which were limited by awareness and the number of food sources accepting SNAP. For example, the higher pricing of the remaining Harris Teeter grocery store in the St. Paul’s area of Norfolk is restrictive since the more affordable Save A Lot grocery store closed. Participants called for additional awareness raising.

Social and physical barriers associated with development density, seasonality, limited operating hours, and flooding were also related to transportation. Walkability is particularly problematic for the elderly, individuals with mobility challenges, and those with families including young children. Traffic volume, speed, and missing crosswalks make crossing roads on foot to access food unsafe as was the case along George Washington Hwy/US 17 in Portsmouth. Social tensions also prevented movement between some neighborhoods, such as the Ghent neighborhood in Norfolk. Street flooding, which can be prolonged at times owing to the region’s low-lying location, can prevent access to food sources for many residents due to restricted walkability and altered bus routes around flooded roadways and sidewalks. Participants called for accessibility assessments.

Bus availability decreased in Portsmouth in recent years due to reduced revenues, causing difficulties, especially for households without cars. One participant reflected, “You have a lot of people in Portsmouth who do not own vehicles necessarily, so transportation is a barrier overall to get to certain places. […] There are some transportation issues that we run into – families, especially, large families.” This was not isolated to Portsmouth. Another participant stated, “Past that East Beach area [of Norfolk] a lot of residents there don’t have cars, so they must carry their groceries over the bridge back into the neighborhood.” Participants suggested car ownership as a means to identify reduced access based on density. Development density either in the form of public housing or downtown areas was also suggested to indicate food insecurity, though the impacts vary by income.

### Agency based recommendations

Connectivity with health, public, and social services and the private sector on food security was instrumental in participant recommendations. Participants noted that capitalizing upon synergistic activities will be critical in expanding partnerships, particularly non-traditional ones. Participants recommended addressing systemic and generational issues with poverty to address healthy food access, incorporating non-traditional spaces into food distribution, incentivizing or regulating healthy food options in stores, increasing collaboration particularly with health providers, and improving educational opportunities for healthy food preferences, preparation, and access. To fully address poverty, participants stated that rents would need to be adjusted, which participants suggested would be better approached as a region instead of city by city. Improving partnerships with large employers, such as the Navy, could fill data gaps and improve wellbeing for enlisted service members that should have appeared in food security risk assessments but did not. More data sharing and referrals were thought to benefit hospitals and the outcomes of their patients. Increased access to food assessments from external partners could improve planning, especially for parallel initiatives.

City and regional interventions were called upon to adjust implementation of national programs and influence economic development and investment. While some participants suggested requiring, incentivizing, or penalizing stores that do not traditionally carry healthy food to do so, others reported that past efforts to do so failed due to costs in maintaining the food and limited demand. A participant stated, “There are ways to put incentives or to force, if you will, on Dollar Generals and things like that to buy—when you apply to build, that the city can impose, you have to have 500 square feet [46.5 m^2^] that’s dedicated to fresh fruit and vegetables. So, there are political ways to get those things into that environment and there are cities that have done this across the nation.” Concerns about food waste were also expressed. A participant stated, “There's enough food out there, it's just getting thrown.” This holistic food systems view required additional advocacy to get stores and restaurants as well as health officials to buy into. Nonprofit involvement was mentioned as a successful way to redistribute food in areas without grocery stores. It may also improve access to culturally appropriate foods based on relationships with minority communities. Participants also determined that individuals that track and realign resources, sometimes from their own funds, based on their awareness of individual and family need in their neighborhood, referred to as ‘community heroes’, are critical to food security and need to be documented. City regulations, regional planning initiatives, business, and personal relationships all feed into these recommendations.

Availability alone will not improve diets. Participants emphasized that change has to be holistic, calling for increased education in schools regarding food preference and cooking, adjustment of educational programs required to receive government issued food benefits to be less demeaning and more convenient, supplementation of fixed location government sponsored programs with mobile training units, reduced barriers to accessing benefits, and advocacy to increase the amount of food sources accepting benefits. From the school-based education lens, a participant suggested the following to improve food preference, “Teach kids in schools how to grow vegetables at home through gardens especially where less available in public schools. Tackle at the elementary school level to get ahead of obesity.” From the food assistance program lens, another participant explained, “We can put efforts in […] communities to do with SNAP education […] You can give me this money but if I don’t know how to shop well then I’m not going to use it well […] There’s that pervasive belief in a lot of our lower-secure communities that these programs are really hard to access.” To make food education more convenient, programming outside the school system was suggested through the Virginia Cooperative Extension at libraries, parks, virtual spaces, or community centers. Other participants believed these programs were too hard to access because typically an individual’s credit had to be destroyed before becoming eligible, which may require program redesign to surmount. Further, collaboration across service sectors was recommended for case management to help individuals navigate food access, resources, and support programs. Relationships with federal programs, local non-profits, school officials at several levels of government, and eligible residents are needed to support these changes.

Participants identified both need for and opportunities to build connectivity between various neighborhood, city, and regional food assistance providers. On the one hand, a participant shared about this work, stating, “We’re actually trying to work with the Foodbank now to see what we can do to help eliminate the barriers for some of these smaller food pantries […] because it’s not as easy as one would hope it would be. Sometimes they [the food pantries] don’t have things. You have to have a pest control review and all sorts of things that they may not have either the finances to do or the knowledge about.” Increased storage for healthy foods and improved healthy food options, beyond cans, was still thought to require additional nonprofit funding and space. Abandoned strip malls near schools were suggested for conversion into foodbanks where students can shop for and take-home meals. On the other hand, another participant shared “What I found is that the libraries are a great hub of information for all these neighborhoods because many of them […] walk to their library [which] were actually getting ready to kick off a food forest and food bank and community garden at one of the libraries in South Norfolk. […] Many of them are due to summer food feeding programs as well.” Fostering such collaborations can improve both resources and connectivity as well as communication. Regarding communication, campaigns were suggested including social media platforms, television and radio commercials. Sponsorships were suggested to fund such media campaigns and other education programming. Participants also suggested combining efforts with diversity training and other ongoing initiatives.

## Discussion

Connections between the spatial data and socio-economic factors inform ways to adjust data collection techniques as well as assessment and implementation frameworks for food security intervention planning. The capacity to index and weight non-traditional sources of food security, such as the community gardens and fridges, mobile or pop-up options in real-time as suggested by study participants, must be developed to inform geo-analytic frameworks [51]. Seven contributors (i.e. availability, proximity, accessibility, awareness, affordability, cooking capacity, and preference) to food security were identified. These align with factors in Mui et al.’s [70] causal loop developed from a community-based workshop in Baltimore, Maryland and expand beyond the USDA food security indicators (i.e. proximity, accessibility, and affordability).

### United States food systems applications

At a United States food systems level, these seven factors address both producer and consumer contributions to nutrition, specifically distribution, acquisition, preparation, and consumption [71]. Participant additions to sources and unmet need as well as recommendations also address known food security failure points from the literature, including resources, awareness, access, and skills associated with individual and organizational capacity and agency. Financial, technological, regulation, and other policies were also identified contributors at the systems level [71]. Cooksey-Stowers et al. [32] and Leslie et al. [72] similarly show that regulatory intervention, for example, addresses systemic issues related to low quality food sources common in low-income, low- mobility areas through shifting the ration of food present in a location or the types of locations present by zoning to restrict unhealthy food and provide healthy options.

Both the food environment and utilization aspects of the food system are represented when compared to the Bureau of Resilience and Food Security’s conceptual framework for food security [73]. Recognizing the connections between these elements of the system is critical to successful change. For example, grocery stores have a minimum square footage for profitable sale of healthy food that may limit investment in an area with restrictive regulations such as those being considered by study participants [30]. These considerations are critical to recommendations associated with similar studies from Baltimore, Maryland calling for supermarket retention, small store, market, including online markets, and urban agriculture connectivity through supply chain and transportation adjustments, and nutritional assistance maximization.

### International food systems applications

Participant proposed strategies could also be compared to international household and country level food security capacity building studies. Some examples are given to show the transferability of results produced using the PGIS information sharing for intervention process. Although all results do not directly link to international food system dynamics, the process used generates area specific results that can and should be contextualized from local to national levels. Ansah et al.’s [74] literature review of household food security building strategies from across the world identifies attitudinal, agronomic production, and financial causal pathways. Findings from Norfolk, Suffolk, and Portsmouth, Virginia regarding the role of availability, affordability, and preference align directly with these household factors. Further, agro-production addresses some accessibility, awareness, and potentially cooking capacity related issues through developing crop and livestock husbandry capacity within the household. Allen and Prosperi’s [75] conceptual model of food system resilience based on systems in Mediterranean countries prioritizes nutritional quality, affordability, dietary balance, and cultural preference of the food supply. Preference and affordability were also directly identified as contributors to food security in Southeast, Virginia. Quality and dietary balance of the food supply also indirectly relate to availability, awareness, distribution (i.e. accessibility and proximity), and possibly cooking capacity of healthy food. Further evidence of interrelated factors, such as attitudinal adjustment and resource redistribution, appears in a study from New Zealand showing that community gardens contribute to health as both a source of nutrition and wellbeing because they are also green space [76]. For communities in Southeast, Virginia experiencing limited access to healthy foods due flooding, gardens may have similar effects upon wellbeing and accessibility by increasing green space and improving drainage.

Béné et al. [77] similarly shows that to achieve systemic change and improve upon the underlying ecological contributors to food insecurity in developing countries that coping behaviors and strategies should be addressed systemically through community level social and infrastructural changes and household level income and asset redistribution, increased cash availability, and psychological support. It is also noted, however, that some social capital interventions, such as social capital and education, can have adverse effects upon long-term food security [77]. Inter-relationships between the seven factors contributing to food security identified in Southeast Virginia generated from household and community level insight to systemic, infrastructural, behavioral, and resource promote change without reinforcing poverty traps and before reaching production tipping points. For example, subject matter experts called for generational poverty and demeaning education programs to be addressed for effective food security intervention and these concerns cross-cut all seven factors. Replicable monitoring methods that ensure aggregate interventions are sensitive to such stressors across multiple dimensions at various scales are needed, particularly by international development agencies that have already applied resilience concepts to their food security portfolios [77].

### Informing interventions with PGIS

PGIS provided a scalable, transferable methodology with which to simultaneously collect discrete data about food sources and identify neighborhood level contributors to unmet need in alignment with existing food security assessment metrics. The nuance required for integrating neighborhood level data collected using PGIS is that food environment and utilization as well as producer and consumer perspectives have to be united in the assessment to collect the appropriate information and make actionable resource and ultimately wellbeing adjustments [69]. Responding to participant requests for real-time data entry options using these methods addresses neighborhood deficiencies in nationally available datasets through curated public input. The addition of availability, preference, cooking capacity, and awareness to traditional data would capture a broader range of issues affecting agency to develop and implement interventions at various levels of governance and within neighborhood networks. However, future researchers considering this webGIS approach should remain cognizant that the ability to make inferences on data at one scale based on data visualized at another scale remains limited [40, 41].

## Conclusions and future research

Findings inform how to adjust data collection techniques and intervention planning considerations. Publicly available and other national datasets may not be inclusive of non-traditional food sources or updated frequently enough to reflect changes associated with closures, expansion, or new programs. Practitioners would benefit from accessible data that can be updated to reflect local knowledge in real-time. Publicly available and national datasets were missing 5.9 percent of total food sources including 15.1 percent of healthy food sources. Food pantries, community gardens and fridges, farmers markets, child and adult care programs, and meals served in community centers and homeless shelters were not well represented. Just under half of participants identified areas of need (24.09 km^2^) fell outside USDA low income, low access areas. Economic, physical, and social barriers to food security were interconnected with transportation limitations in Southeastern Virginia. Recommendations addressed systemic and generational issues with poverty, incorporating non-traditional spaces into food distribution systems, incentivizing or regulating healthy food options in stores, increasing data sharing, and improving educational opportunities. City and occasionally regional level intervention needs to be conducted strategically to adjust implementation of national programs, influence economic development and investment, and recognize ‘community heroes.’ Capitalizing upon synergistic activities will be critical in expanding partnerships to achieve these goals, particularly non-traditional ones.

To address neighborhood scale food security needs, data collection and assessment should address both environment and utilization issues and consumer and producer perspectives including availability, proximity, accessibility, awareness, affordability, cooking capacity, and preference. These seven contributors to food security relate to findings from other cities in the United States. Further, they align with some aspects of national food system dynamics in the United States and other countries. While the same set of indicators may not be applicable outside the mid-Atlantic region, the PGIS process utilized to facilitate information sharing about neighborhood level contributors to food insecurity and translate those contributors to intervention strategies through discussion with local subject matter experts and contextualization within larger scale food systems dynamics is transferable. This web based and online content has growing potential to improve data collection and assessment by complementing national datasets with neighborhood input as engagement and utilization opportunities increase with internet access proliferation.

Pilot studies, such as this, serve as a means with which to validate micro-simulation techniques utilized to downscale national food security related datasets and train data collection models to compile and weight locally known and relevant information for decision making. Similar studies could be conducted to test national data sets on store and service type contributions to food security and shopping behavior. Future research should include perspectives from households experiencing food insecurity to validate stakeholder and federal data using methods like the Community Assessment for Public Health Emergency Response, a rapid epidemiological assessment used to conduct household interviews in a quick serpentine succession [78], that can feed into mapping tools or by engagement in a combined or parallel participatory mapping process. Additional research is needed on the “edge effect” to identify why some stakeholder identified areas of need were outside USDA identified areas and to what extent the whole USDA area was in need according to stakeholders. The utility of other vulnerability indices in predicting food security should also be analyzed.

Further analysis could unite stakeholder-informed GIS models with GIS-based multicriteria decision analysis, a commonly applied technique in urban planning and analyzing spatial decision problems, to delimit target areas for health interventions. Such an approach could capture dynamic urban changes, incorporate objective demographic, economic and health data as well as the additional input and factor weighting from subject matter experts. Health data should also be included in future research to directly identify the impacts and contributors thereof to food insecurity.

## Data Availability

All data generated or analyzed during this study are included in this published article [and its supplementary information files – see Table 1].

## Abbreviations

GIS: Geographic information systems
SNAP: Supplemental Nutrition Assistance Program
USDA: United States Department of Agriculture
PGIS: Participatory geographic information systems
HFAI: Healthy Food Availability Index
LIFE: Location Intelligence for Food Equity
CDC: Center for Disease Control and Prevention
HRGEO: Hampton Roads Geospatial Exchange Online
CACFP: Childhood Adult Care Food Program
MAUP: Modifiable Areal Unit Problem
